# Formononetin promotes porcine oocytes maturation and improves embryonic development by reducing oxidative stress

**DOI:** 10.3389/fcell.2024.1520429

**Published:** 2025-01-09

**Authors:** Na Wang, Han Yang, Yelei Chen, Hekun Wang, Chaorui Wang, Jianglin Fan, Yajie Chen, Yinghua Li, Maobi Zhu

**Affiliations:** ^1^ Guangdong Provincial Key Laboratory of Large Animal Models for Biomedicine, South China Institute of Large Animal Models for Biomedicine, School of Pharmacy and Food Engineering, Wuyi University, Jiangmen, China; ^2^ Department of Gynecology, Jiangmen Maternity and Child Healthcare Hospital, Jiangmen, Guangdong, China

**Keywords:** formononetin, mitochondrial function, oxidative stress, Nrf2/Keap1, autophagy

## Abstract

Increasing evidence has demonstrated that oxidative stress impairs oocyte maturation and embryonic development. Conventionally, antioxidants have been applied *in vitro* systems to improve oocyte maturation and blastocyst rates. Formononetin (FMN) is a flavonoid that has been shown to have various pharmacological effects, including antioxidants. In this study, we delved into the impact of FMN, acting as an antioxidant, on the *in vitro* development of oocytes and blastocysts within the culture system. FMN supplementation at 0.5 μM enhanced the rate of first polar body extrusion and blastocyst formation post parthenogenetic activation. It also increased mitochondrial function and ATP levels, reduced intracellular reactive oxygen species, and elevated intracellular GSH levels in both oocytes and embryos. Moreover, FMN significantly decreased autophagy and apoptosis levels in blastocyst cells, potentially via regulation of the Nrf2/Keap1 pathway. This is the first study to report that FMN supplementation benefits the *in vitro* culture of oocytes and early embryo development, potentially by regulating oxidative stress, mitochondrial function, and autophagy.

## Introduction

*In vitro* oocyte maturation (IVM), *in vitro* fertilization (IVF), and *in vitro* embryo culture (IVC) techniques have been widely used in agricultural and biomedical research (Gil et al., 2010). Especially, these techniques serve as the basis of modern biomedical assisted reproductive technologies, such as assisted reproductive technology (ART), somatic cell nuclear transfer (SCNT), and embryo cryopreservation (Grupen, 2014). However, external factors associated with a high-oxygen environment and *in vitro* procedures can adversely affect oocyte quality and suppress embryonic development.

During the process of oocyte and embryo *in vitro*, unstable extracellular environments, such as excessive oxygen stress, light, water, oocyte handling, culture medium composition (Ciray et al., 2009; Bennemann et al., 2018; Lee et al., 2017; Li et al., 2015), and other uncertain factors, could impair oocyte maturation and embryo development. Excessive ROS causes a series of injuries to oocytes and embryos, such as induction of mitochondrial dysfunction, DNA fragmentation, apoptosis, and cytoplasmic abnormalities (Amin et al., 2014; Agarwal et al., 2005; Agarwal et al., 2006). The use of substances with antioxidant properties reducing excessive ROS could ameliorate oocyte quality and enhance embryo developmental competence *in vitro*. Therefore, exploring the role of antioxidants in oxidative stress during oocyte and embryo *in vitro* is crucial. Meanwhile, it is also beneficial to the treatment of woman infertility (Li et al., 2019).

Formononetin (7-hydroxy, 4′-methoxy isoflavone, FMN) is a representative flavonoid found in Chinese medicinal plants, such as Red Clover, Astragalus, Licorice, and Pueraria (Machado Dutra et al., 2021). FMN has been reported to have a variety of biological activities. Such as antioxidant, anti-inflammatory, anti-allergic, neuroprotective, anti-hypertensive, and anti-cancer (Li et al., 2018; Sun et al., 2011; Mu et al., 2009; Tian et al., 2013; Chen et al., 2014). Moreover, previous studies have shown that FMN is a kind of phytoestrogens of relevant interest in the areas of nutrition, medicine, and cosmetics can be used in hormone replacement therapy (Kulling et al., 2002; Dornstauder et al., 2001), which indicates it may have potential benefits for female and reproductive health. FMN exhibits strong free radical scavenging activity and is attributed to their influence on the antioxidant enzymes and non-enzymatic system by scavenging free radical (Mu et al., 2009).

Although FMN is a flavonoid in herbal medicine and has well-known protective properties, the role of FMN in mammalian oocytes and embryos has not been studied. Our study aimed to explore the benefits of FMN on oocyte maturation and early embryonic development. Oocytes and embryos were exposed to FMN and evaluated oocyte first polar body extrusion rate, blastocyst rate, ROS, apoptosis, mitochondrial function, and autophagy.

## Materials and methods

### Animals and chemicals

Unless otherwise stated, all reagents and chemicals were purchased from Sigma-Aldrich (St. Louis, MO, United States). Porcine ovaries used in this study were obtained from a slaughterhouse (Jiangmen, China). Formononetin (Selleck) was dissolved in DMSO to make a 50 mM stock solution. Different concentrations of FMN were tested *in vitro* maturation (IVM) or *in vitro* culture (IVC) media to assess their dose-dependent effects.

### Oocyte retrieval and *in vitro* maturation

Cumulus–oocyte complexes were collected from 3- to 8-mm follicles. Oocytes with a minimum of three layers of cumulus cells were selected with a stereomicroscope and cultured *in vitro* in maturation medium (IVM; M199 medium with 100 μL penicillin-streptomycin, 1 mL porcine follicular fluid, 0.2 mg epidermal growth factor, 0.22 mg sodium pyruvate, 100 IU luteinizing hormone, 100 IU follicle stimulating hormone, and 0.9 mg L-cysteine) for 44 h at 38.5°C in an incubator of 5% CO_2_ and 100% humidity.

### Parthenogenetic activation and *in vitro* embryo culture

At the end of maturation, COCs were removed from IVM and were repeatedly pipetted with 0.25% hyaluronidase. Oocytes with polar bodies were activated by 120 V direct current stimulation twice, each time for 60 μs followed by 0.1 s of pause using an electro cell fusion generator and transferred to 7.5 mg/mL cytochalasin B to balance for 3 h. Finally, they were cultured in bicarbonate-buffered porcine zygote medium 5 (PZM- 5) with 4 mg/mL BSA for 7 days at 38.5°C, 5% CO_2_, and 100% humidity.

### Measurement of intracellular ROS and GSH levels

MII-stage oocytes and 4-cell stage embryos were cultured with 10 µM 2′,7′-dichlorodihydrofluorescein diacetate (DCFH, #S0033, Beyotime, Shanghai, China) or 10 µM 4-chloromethyl-6,8-difluoro-7-hydroxycoumarin (CMF2HC, #C12881, Invitrogen,) at 37°C for 45 min and 30 min in darkness, respectively. Fluorescence intensity was analyzed using a fluorescence microscope and ImageJ software.

### Oocyte mitochondrial abundance assessment

Oocytes were cultured with 100 ng/mL Mito-Tracker Red CMXRos (#C1049B Beyotime, Shanghai, China) for 30 min in darkness. Oocytes were rinsed thrice and photographed by fluorescence microscope. Fluorescence intensity was analyzed using a fluorescence microscope and ImageJ software.

### Embryo mitochondrial membrane potential (MMP, Dψm) assay

Embryos were cultured with 10 µM 5,5′,6,6′-tetrachloro-1,1′,3,3′-tetraethylbenzimidazolylcarbocyanineiodide (JC-1; #C2006, Invitrogen) for 1 h in darkness, with red fluorescence indicating the aggregated form (J-aggregate) and green fluorescence indicating the monomer form (J-monomer). The red/green fluorescence intensity was used to calculate the levels of MMP.

### 5-Ethynyl-2′-deoxyuridine (EdU) assay

In brief, blastocysts were cultured in 5 μM EdU (#C10310-1, Ribobio, China) for 8 h at 38.5°C. Subsequently, the blastocysts were fixed in 4% paraformaldehyde and permeabilized using 0.3% Triton X-100% and 5% fetal bovine serum for 30 min. The detection of EdU was performed according to the manufacturer’s protocol and the number of fluorescent cells was analyzed using a fluorescence microscope and ImageJ software. The ratio of the number of EdU-positive nuclei to the total number of nuclei was used to compute the cell proliferation rate.

### Immunofluorescence staining

After being fixed with 4% paraformaldehyde, blastocysts were permeabilized and blocked with 0.3% Triton X-100% and 5% fetal bovine serum for 30 min. Next, blastocysts were treated with primary antibodies to LC3B antibody (1:200; #ab48394, Abcam) and anti-NRF2 antibody (1:200; #ab31163, Abcam) overnight at 4°C. After washing, blastocysts were incubated with corresponding secondary antibodies for 1 h. TUNEL staining was carried out using the Situ Cell Death Detection kit (#11684795910, Roche) according to the manufacturer’s instructions. Nuclei were counterstained with DAPI (#62248, Thermo Fisher Scientific). Finally, fluorescent microscope and ImageJ software were used to analyze the fluorescence intensity.

### Determination of ATP levels

ATP in 4-cell stage embryos was measured using an ATP detection kit (#S0026, Beyotime, China). 4-cell stage embryos were lysed and the supernatant was collected. Then, supernatant and ATP working solution were mixed and analyzed using a multifunctional enzyme marker (Synergy Neo2, BioTek) via chemiluminescence.

### Western blot analysis

60 blastocysts were lysed with RIPA buffer (#89900, Thermo Fisher Scientific) containing a protease inhibitor cocktail. The protein samples were separated by 8% SDS-polyacrylamide gel and wet-transferred to a PVDF membrane at 200 mA and 4°C for 2 h. The membrane was blocked using 5% bovine serum albumin with Tween 20 (TBS-T), followed by the incubation with primary antibodies overnight at 4°C. And incubation with horseradish peroxidase-conjugated secondary antibodies at RT for 1 h. Primary antibodies were rabbit monoclonal antibodies against NRF2 (1:2000, #16396-1-AP, Proteintech), KEAP1 (1:2000, #10503-2-AP, Proteintech), and β-Actin (1:5000, #20536-1-AP, Proteintech). Secondary antibodies were goat anti-rabbit IgG (#31460, Thermo Fisher Scientific). Signals were developed using an enhanced chemiluminescence substrate (#34580, Thermo Fisher Scientific), and images were acquired using an Azure Sapphire RGBNIR detection system.

### Quantitative real-time reverse transcription-polymerase chain reaction (qRT-PCR)

Total RNA was extracted using the Dynabeads mRNA DIRECT Purification Kit (#61012, Invitrogen) and reverse transcribe the RNA into cDNA using the iScript cDNA Synthesis Kit (#1708891, iScript cDNA Synthesis, BIO-RAD). qRT-PCR was performed using KAPA SYBR FAST Universal qPCR Kit (#KK4601, Kapa Biosystems, United States) and LightCycle96 devices (Roche) (Liu et al., 2023). The 2^−ΔΔCt^ method were used to quantify the mRNA expression of genes, and GAPDH was used as the standard. The primer sequences are listed in Table 1.

**TABLE 1:** Information about the primer sequences.

	Primer sequences (5′–3′)		Anneal temperature (°C)
	Forward	Reverse	
GAPDH	TTCCACGGCACAGTCAAG	ATA​CTC​AGC​ACC​AGC​ATC​G	60
PTX3	GGC​CAG​GGA​TGA​ATT​TTA​C	GCT​ATC​CTC​TCC​AAC​AAG​TGA	60
HAS	TGG​CTG​TAC​AAT​GCG​ATG​TG	TGG​GTG​GTG​TGA​TTT​TCA​CC	60
PTGS1	AAC​ACG​GCA​CAC​GAC​TAC​A	CTG​CTT​CTT​CCC​TTT​GGT​CC	60
PTGS2	ACA​GGG​CCA​TGG​GGT​GGA​CT	CCA​CGG​CAA​AGC​GGA​GGT​GT	60
CD44	GAG​GAT​GAT​ATG​AGC​AGT​GG	GGT​GCG​TAG​TAG​TCG​GAA​G	60
CASP8	GCC​TCG​GGG​ATA​CTG​TTT​GA	CGC​TGC​ATC​CAA​GTC​TGT​TC	60
CASP3	GAC​GGA​CAG​TGG​GAC​TGA​AGA	GCC​AGG​AAT​AGT​AAC​CAG​GTG​C	60
BCL2	GCCGAAATGTTTGCTGAC	GCCGATCTCGAAGGAAGT	60
GPX	GGT​CTC​CAG​TGT​GTC​GCA​AT	TCG​ATG​GTC​AGA​AAG​CGA​CG	60
PGC1α	TTC​CGT​ATC​ACC​ACC​CAA​AT	ATC​TAC​TGC​CTG​GGG​ACC​TT	60
PRDX2	TGT​CCT​TCG​CCA​GAT​CAC​T	TCCACGTTGGGCTTGATT	60
CAT	AGC​CAG​TGA​CCA​GAT​GAA​GCA​TTG	ATG​TCG​TGT​GTG​ACC​TCA​AAG​TAG​C	60
SOD1	GTT​GGA​GAC​CTG​GGC​AAT​GT	CGG​CCA​ATG​ATG​GAA​TGG​TC	60
SOD2	AAT​CTG​AGC​CCT​AAC​GGT​GG	GAC​GGA​TAC​AGC​GGT​CAA​CT	60
NOX2	TGT​ATC​TGT​GTG​AGA​GGC​TGG​TG	CGGGACGCTTGACGAAA	60
ATP5B	TTGTTGGCAGTGAGCATT	AACCTGGAATGGCTGAGA	60
TFAM	CGC​TCT​CCG​TTC​AGT​TTT​GC	TGC​ATC​TGG​GTT​CTG​AGC​TTT	60
NRF1	CCT​GTG​AGC​ATG​TAC​CAG​ACT	ACT​GTT​CCA​ACG​TCA​CCA​CCT	60
NRF2	AGC​GGA​TTG​CTC​GTA​GAC​AG	TTC​AGT​CGC​TTC​ACG​TCG​G	60
ATG5	TTG​CAG​TGG​CTG​AGT​GAA​CA	TCA​ATC​TGT​TGG​TTG​CGG​GA	60
P62	AAG​AAC​GTA​GGG​GAG​AGT​GTG	TTC​CCT​CCA​TGT​TCC​ACG​TC	60
BECLIN1	AGG​AGC​TGC​CGT​TGT​ACT​GTT​CT	TGC​TGC​ACA​CAG​TCC​AGG​AA	60
LC3	TTC​AAA​CAG​CGC​CGA​ACC​TT	TTT​GGT​AGG​ATG​CTG​CTC​TCG	60
UCHL1	ACT​TTG​GAT​TCG​CTC​GGT​AC	CGC​TTA​TCT​GCA​GAC​CCC​AA	60

### Statistical analysis

Statistical analysis was performed with the SPSS and Graphpad Prism. One-way ANOVA and Duncan’s tests were used to process data. Data were presented by average ±SEM. Different letters (*p* < 0.05) denoted significant differences. There were at least three biological replicates in each group.

## Results

### FMN promotes porcine oocyte maturation and embryonic development

FMN has been shown to have antioxidant and anti-inflammatory effects at concentration of 10 μM, particularly inhibiting Aβ-induced vascular inflammation (Fan et al., 2022). In our initial experiments, we designed a concentration range of 0.5–50 μM (Supplementary Data). However, we found that concentrations exceeding 10 μM inhibited oocyte polar body extrusion and blastocyst formation. On the other hand, the addition of 0.5 μM FMN to the IVM and IVC media significantly increased the rate of polar body extrusion at the MII stage (a: with polar body, b: without polar body) and the blastocyst formation rate after parthenogenetic activation.

**FIGURE 1:**
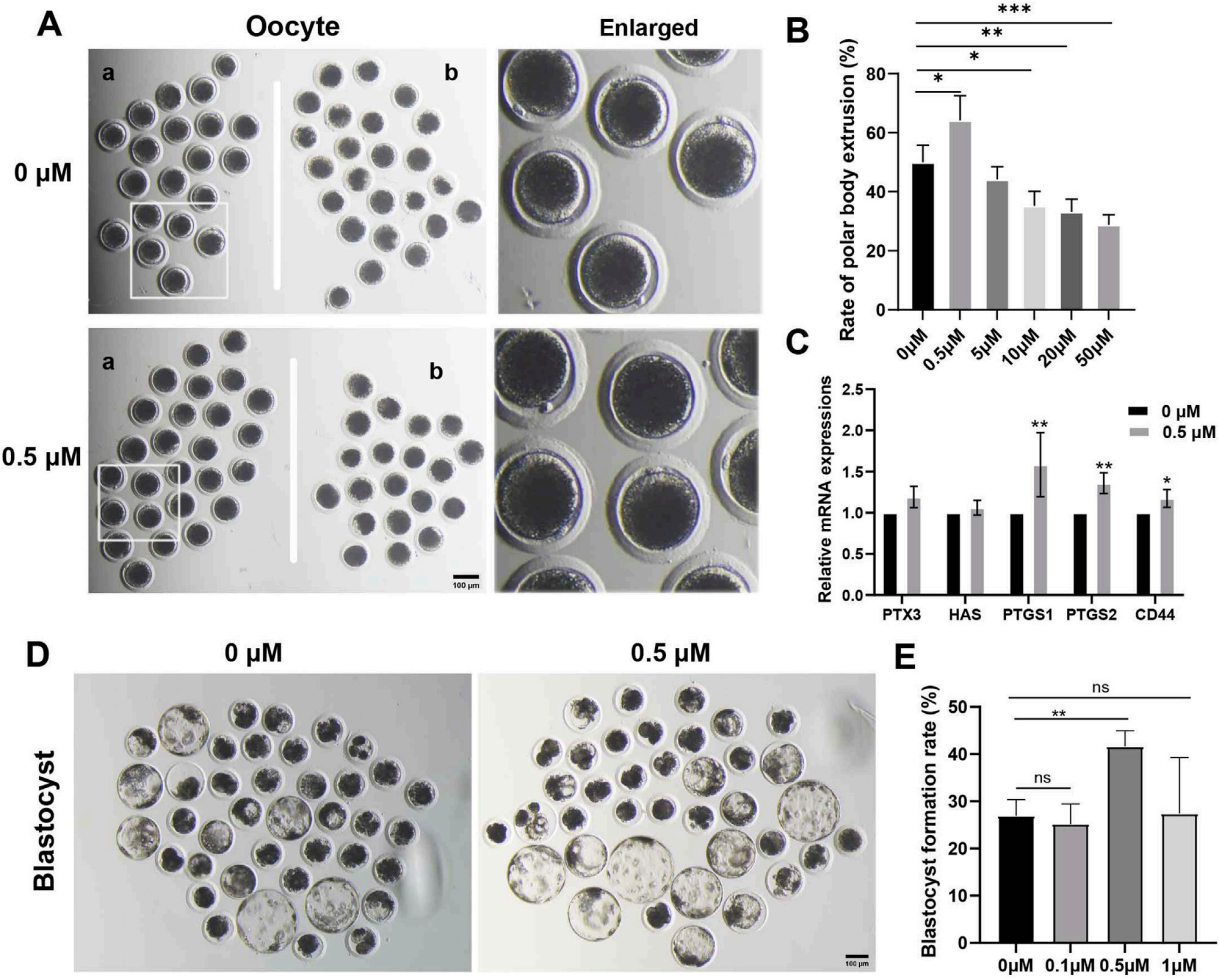
Effects of FMN on the oocyte polar body extrusion and blastocysts development *in vitro*. **(A)** Oocyte polar body extrusion in the control and FMNtreated groups. **(a)** Oocytes with polar body, **(b)** oocytes without polar body, scale bar, 100 μm. **(B)** The rate of different concentrations of FMN treatment on the polar body extrusion in porcine oocytes **(C)** Relative mRNA levels of cumulus expansion related genes PTX3, HAS2, PTGS2, PTGS1, and CD44 in oocytes. **(D)** Embryonic development rate on day 7 in the control and FMN-treated groups, scale bar, 100 μm. **(E)** The rate of blastocyst formation under treatment of various concentrations of FMN. Data are presented as the mean ± standard deviation (SD). *p < 0.05; **p < 0.01, ***p < 0.001 vs. 0 μM FMN group.

During *in vitro* oocyte maturation, the rates of polar body extrusion in the FMN treatment groups (0, 0.5, 5, 10, 20, and 50 μM,) were (50.05% ± 5.73%, 64.22% ± 8.37%, 44.16% ± 4.31%, 35.21% ± 4.89%, 33.22% ± 4.22%, and 28.81% ± 3.40%, respectively; Figures 1A, B). Furthermore, we examined genes associated with cumulus expansion, including pentraxin-3 (PTX3), hyaluronan synthase 2 (HAS2), prostaglandin endoperoxide synthase 2 (PTGS2), prostaglandin endoperoxide synthase 1 (PTGS1) and hyaluronan receptor (CD44), among which PTGS2, PTGS1, and CD44 were significantly increased compared to the control (1.59 ± 0.16, 1.36 ± 0.07, 1.12 ± 0.05, respectively; Figure 1C).

During *in vitro* embryo cultivation, the blastocyst rates in the FMN treatment groups (0, 0.1, 0.5, and 1 μM) were (26.95% ± 3.40%, 25.18% ± 4.27%, 41.63% ± 3.31% and 27.45% ± 11.83%, respectively Figures 1D, E). In conclusion, the group treated with 0.5 μM FMN showed a significant increase in the rate of blastocyst formation.

### FMN augments the antioxidant activity of porcine oocytes and early embryos

To explore whether FMN promotes oocyte maturation and embryonic development in association with the antioxidant properties, we measured the levels of ROS and GSH, an endogenous antioxidant, in porcine MII stage oocytes and 4-cell stage embryos [a critical period for porcine early embryo zygotic genome activation (ZGA)] (Liu et al., 2023), respectively.

**FIGURE 2:**
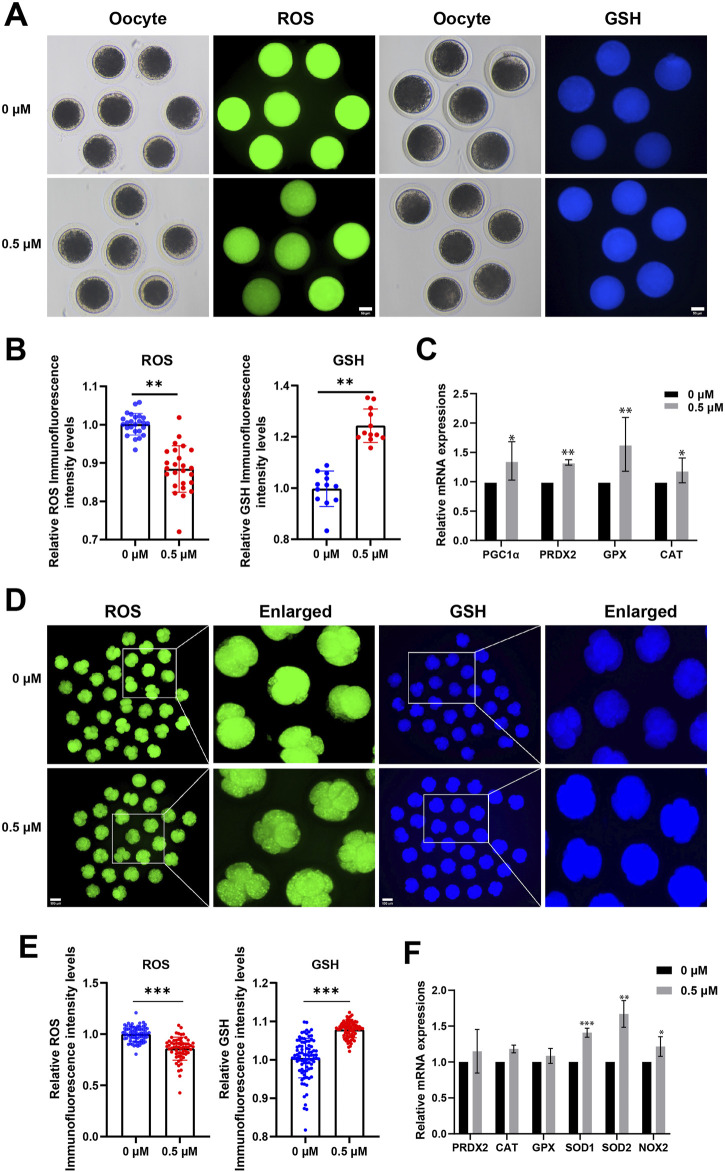
Effect of FMN on the antioxidant capacity of oocytes and early embryos. **(A)** Oocyte ROS (green) and GSH (blue) expression at MII stage in control and FMN-treated groups, scale bar, 50 μm. **(B)** Relative fluorescence intensity levels of ROS were analyzed for control and FMN-treated group. **(C)** Relative mRNA levels of antioxidant-related genes PGC1α, PRDX2, GPX and CAT in oocytes treated with or without FMN. **(D)** Embryonic ROS (green) and GSH (blue) expression in 4-cell stage in control and FMN-treated groups, scale bar, 100 μm. **(E)** Relative fluorescence intensity levels of embryonic ROS and GSH were analyzed for control and FMN-treated group. **(F)**: Relative mRNA levels of antioxidant-related genes PRDX2, CAT, GPX, SOD1, SOD2 and NOX2 in embryos treated with or without FMN. Data were presented as the mean ± standard deviation (SD). *p < 0.05,**p < 0.01, ***p < 0.001 vs. 0 μM FMN group.

In MII stage oocytes, comparison to the control group, the FMN-treated group’s fluorescence intensity of ROS was significantly lower (0.88 ± 0.06), whereas the fluorescence intensity of GSH was significantly higher (1.24 ± 0.06; Figures 2A, B). Antioxidant-related genes peroxisome proliferator-activated receptor γ coactivator 1-alpha (PGC1α), peroxiredoxin 2 (PRDX2), glutathione peroxidase (GPX), and catalase (CAT), were also higher than those in the control group (1.36 ± 0.33, 1.32 ± 0.05, 1.64 ± 0.46, and 1.20 ± 0.21, respectively Figure 2C).

In 4-cell stage embryos comparison to the control group, the FMN-treated group’s fluorescence intensity of ROS was significantly lower (0.86 ± 0.11), whereas the fluorescence intensity of GSH was significantly higher (1.37 ± 0.05; Figures 2D, E). Antioxidant-related genes peroxisome superoxide dismutase 1 (SOD1), superoxide dismutase 2 (SOD2), and oxidative stress-related gene NADPH oxidase 2 (NOX2)—were more significantly elevated in the FMN-treated group (1.40 ± 0.06, 1.67 ± 0.19, and 1.22 ± 0.14, respectively Figure 2F). This suggests that FMN can boost the antioxidant activity of porcine oocytes and early embryos, mitigating ROS, and increasing GSH levels.

### FMN enhances mitochondrial function of porcine oocyte and early embryos

As mitochondrial dysfunction can significantly contribute to increased ROS levels and hinder oocyte and embryo development, we used Mito-Tracker for MII-stage oocytes and JC-1 for 4-cell stage porcine embryos to assess their mitochondrial function.

**FIGURE 3:**
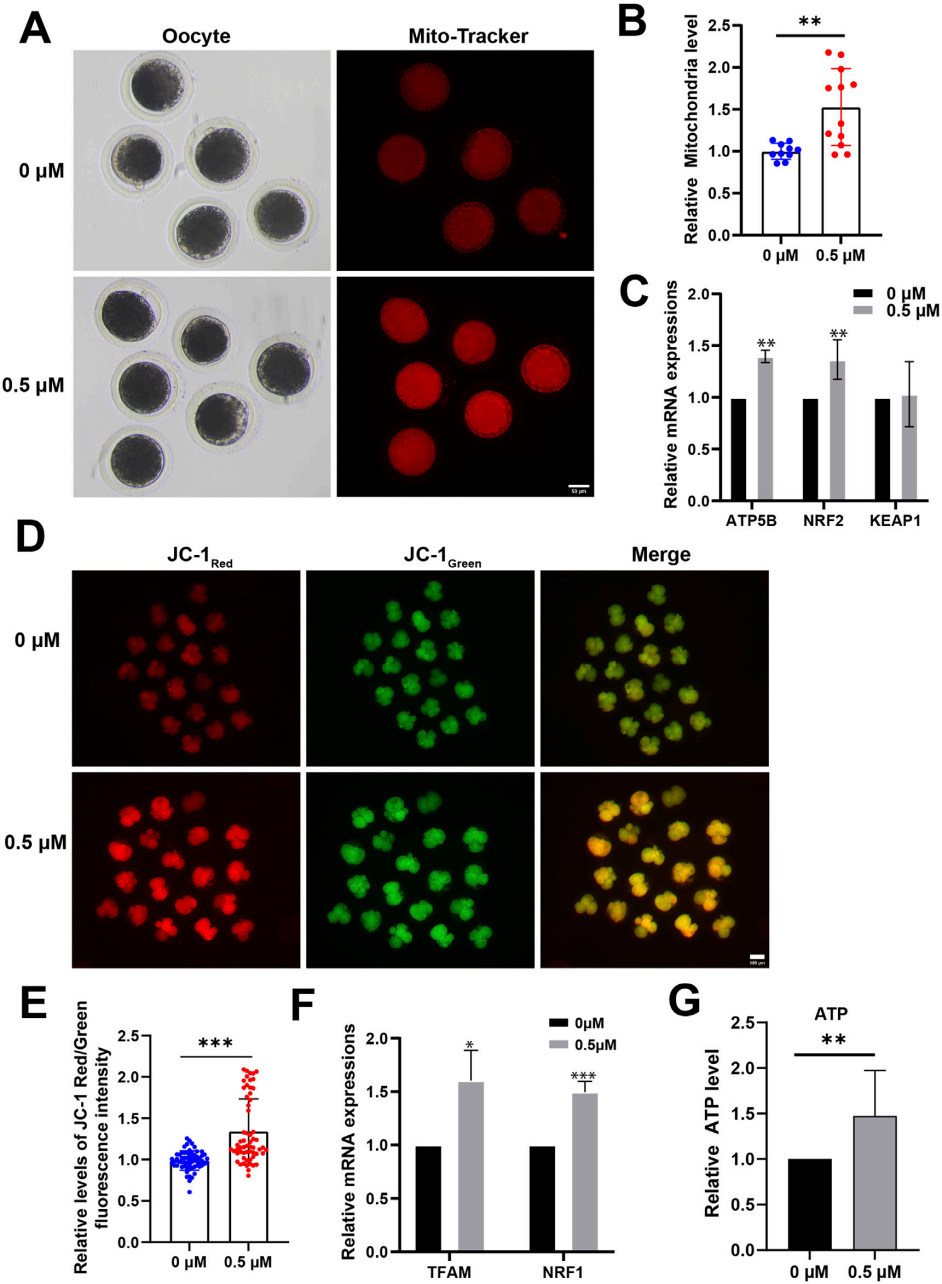
Effect of FMN on mitochondrial function in oocytes and early embryos. **(A)** The mitochondria distribution at MII stage were stained with Mito-Tracker Red; scale bar, 50 μm. **(B)** The relative abundance of mitochondria in oocyte were analyzed for control and FMN-treated group. **(C)** Relative mRNA levels of ATP5B, NRF2 and KEAP1 in oocytes. **(D)** 4-cell stage embryos stained with JC-1. Scale bar, 100 μm. **(E)** Relative levels of JC-1 Red/Green fluorescence intensity in embryos were analyzed for control and FMN-treated group. **(F)** Relative mRNA levels of TFAM and NRF1 in embryos. **(G)** ATP levels of 4-cell stage embryos in both control and FMN-treated groups. Data are presented as the mean ± standard deviation (SD). *p < 0.05,**p < 0.01, ***p < 0.001 vs. 0 μM FMN group.

The results show a significant increase in the functional mitochondrial fluorescence levels of MII-stage oocytes in the FMN treatment group compared to the control group (1.53 ± 0.46-fold higher; Figures 3A, B). In addition, the expression levels of mitochondrial function-related genes, ATP synthase F1 subunit beta (ATP5β) and transcription factor nuclear factor erythroid 2-related factor 2 (NRF2), were elevated in FMN treatment group (1.40 ± 0.06; 1.37 ± 0.19, respectively; Figure 3C).

In 4-cell stage embryos, comparison to the control group JC-1 Red/Green fluorescence intensity in FMN-treated embryos was 1.32 ± 5.20-fold higher (Figures 3D, E) and FMN significantly increased the MMP. In addition, the mRNA levels of mitochondrial biogenesis-related genes mitochondrial transcription factor A (TFAM), and nuclear respiratory factor 1 (NRF1) were elevated in the embryos treated with FMN (1.64 ± 0.28 and 1.50 ± 0.10, respectively; Figure 3F). In addition, the ATP levels of 4-cell stage embryos were measured. The results showed a 1.47 ± 0.20-fold increase in FMN-treated embryos compared to the control group (Figure 3G).

### FMN may regulates antioxidant activity and improves mitochondrial function in early embryos via the Nrf2/Keap1 pathway

Next, we investigated how FMN regulates antioxidant activity and improves mitochondrial function in embryos. The transcription factor NF-E2P45-related factor 2 (Nrf2) has been reported to regulate oxidative damage in cells by expressing genes related to the oxidative stress response. It also protects mitochondrial function, thereby regulating cell adaptation and survival under stress conditions (Bellezza, 2018; He et al., 2020; Fang et al., 2020).

**FIGURE 4:**
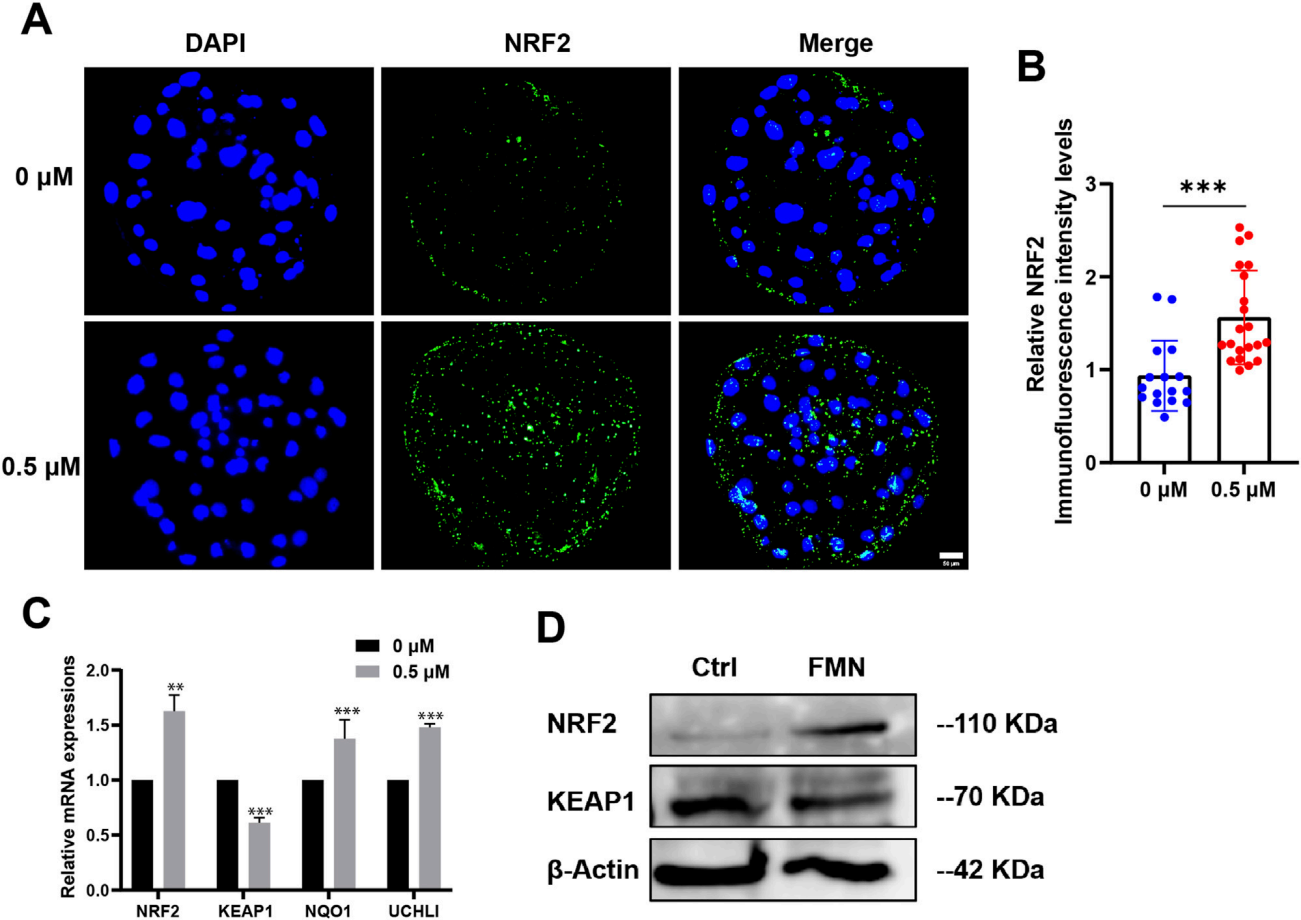
Effect of FMN on the expression levels of the Nrf2/Keap1 signaling pathway-related protein and genes in porcine blastocysts. **(A)** Porcine blastocysts incubated with or without FMN were stained with NRF2 (green) and DAPI (blue), scale bar, 50 μm. **(B)** Relative levels of NRF2 fluorescence intensity in blastocysts were analyzed for the control and FMN-treated group. **(C)** Relative mRNA expression levels of genes related to Nrf2/Keap1 pathway, NRF2, KEAP1, NQO1, UCHL1 in blastocysts. **(D)** Western blot analysis of NRF2 and KEAP1 protein expressions in blastocysts in the control and FMN-treated groups. Data were presented as the mean ± standard deviation (SD). **p < 0.01,***p < 0.001 vs. 0 μM FMN group.

On the day 7, we collected blastocysts for immunofluorescence staining. The results revealed a significant 1.63 ± 15.10-fold enhancement in the immunofluorescence staining of Nrf2 in blastocysts from the FMN-treated group compared to the control group (Figures 4A, B). A recent study suggests that FMN disrupts the dissociation of Nrf2/Keap1 by selectively binding to Keap1. This disruption increases the nuclear expression of Nrf2, potentially addressing oxidative stress-induced peripheral neuropathy and mitochondrial dysfunction (Fang et al., 2020). In addition, we also evaluated the levels of the mRNA and protein associated with the Nrf2/Keap1 pathway. As expected, FMN significantly increased Nrf2 levels while decreasing Keap1 levels (Figures 4C, D).

### FMN reduces autophagy levels in early porcine embryo

**FIGURE 5:**
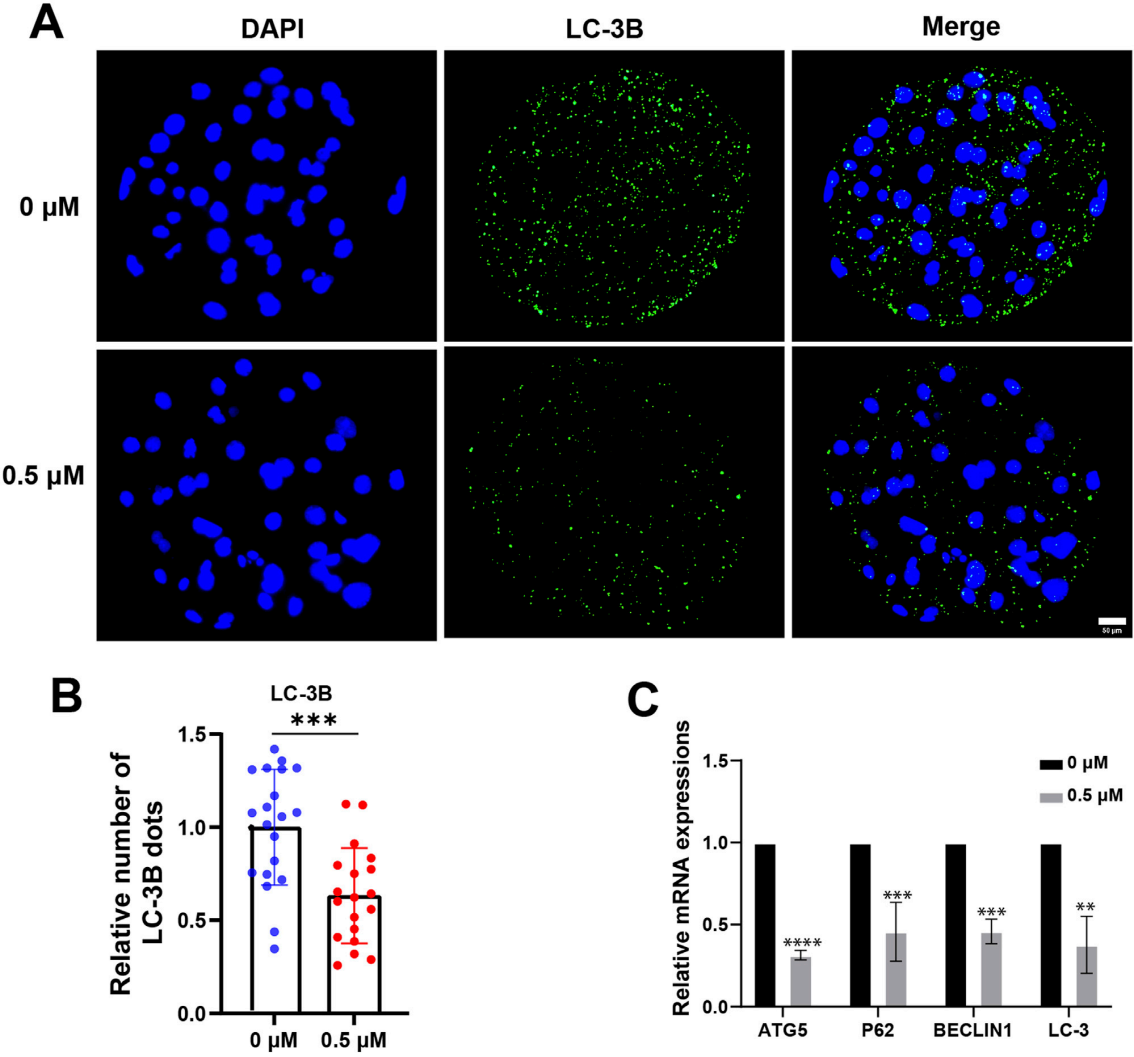
Effect of FMN on autophagy in porcine blastocysts. **(A)** Porcine blastocysts were stained with LC-3B (green) and DAPI (blue), scale bar, 50 μm. **(B)** Relative levels of LC-3B fluorescence intensity in blastocysts were analyzed for the control and FMN-treated group. **(C)** Relative mRNA expression levels of genes related to autophagy ATG5, P62, BECLIN1and LC-3 in blastocysts. Data were presented as the mean ± standard deviation (SD). **p < 0.01, ***p < 0.001, ****p < 0.0001 vs. 0 μM FMN group.

An increasing number of studies have demonstrated crosstalk between the Nrf2 pathway and autophagy, and ROS stress generated by mitochondria can induce autophagy (Scherz-Shouval and Elazar, 2011; Bartolini et al., 2018). We collected blastocysts, examined the levels of the autophagy marker LC3B and assessed the expression of autophagy-related genes. After FMN treatment, the relative fluorescence intensity of LC-3B decreased significantly to 0.89 ± 0.11 (Figures 5A, B). The expression levels of autophagy-related genes ATG5, P62, BECLIN1 and LC3 were also reduced in the FMN-treated group (0.31 ± 0.18, 0.46 ± 0.03, 0.46 ± 0.07, and 0.38 ± 0.17, respectively; Figure 5C).

### FMN can increase the cell proliferation rate and reduce the apoptosis rate of early embryos

**FIGURE 6:**
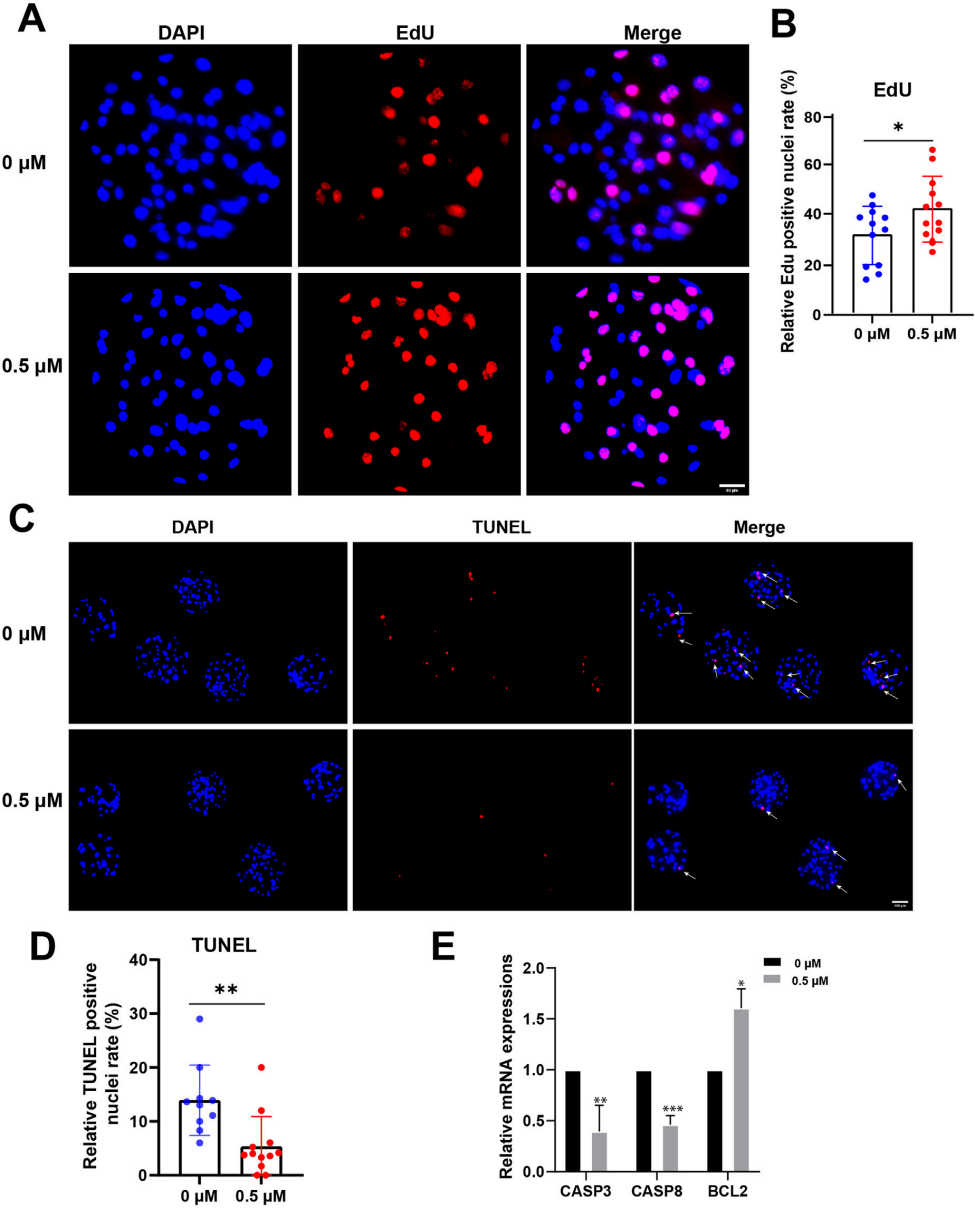
Effect of FMN on proliferation and apoptosis in early embryos. **(A)** Porcine blastocysts were stained with EdU (red) and DAPI (blue), scale bar, 50 μm. **(B)** The rate of EdU-positive cells in blastocysts with control and FMN-treated group. **(C)** Porcine blastocysts were stained with TUNEL (red) and DAPI (blue), scale bar, 100 μm. **(D)** The rate of TUNEL-positive nuclei in blastocysts with control and FMN-treated group. **(E)** Relative mRNA expression levels of CASP3, CASPS8, and BCL2 genes related to anti-apoptosis in blastocysts. Data were presented as the mean ± standard deviation (SD). *p < 0.05, **p < 0.01, ***p < 0.001 vs. 0 μM FMN group.

In summary, we assessed blastocyst quality by examining the proliferation and apoptosis of embryonic cells. On day 7, blastocysts were collected, proliferation and apoptosis of blastocysts were detected by EdU and TUNEL assay respectively. The proportions of EdU-positive nuclei in the control and FMN-treated groups were 31.68% ± 11.33% and 42.29% ± 12.85%, respectively (Figures 6A, B). The proportions of TUNEL-positive nuclei in the control and FMN-treated groups were 12.26% ± 4.03% and 3.99% ± 3.28%, respectively (Figures 6C, D). Additionally, FMN treatment also upregulated the expression of the anti-apoptotic gene BCL2 (1.61% ± 9.34%) but downregulated the expression of the apoptosis-promoting genes Caspase 3 (CASP3) and Caspase 8 (CASP8) (0.60 ± 0.11, 0.54 ± 0.43, respectively; (Figure 6E).

## Discussion

In this study, we confirmed that FMN significantly increased the polar body extrusion of oocytes and early embryo development *in vitro*. FMN is a flavonoid that can be found in herbal dietary supplements like Astragalus and safflower (Machado Dutra et al., 2021). Natural flavonoid compounds, due to their antioxidant activity, have drawn extensive attention for their role in promoting oocyte development. Similar studies have shown that quercetin (Kang et al., 2016; Kang et al., 2013), kaempferol (Zhao et al., 2020), and isorhamnetin (Li et al., 2024) promoted porcine oocyte maturation *in vitro* by reducing oxidative stress and improving mitochondrial functions or increased the cleavage and blastocyst rate of porcine embryos through antioxidant activity. Indicating that the promotion of oocyte maturation and embryo development by FMN may be related to its antioxidant capacity.

The cumulus cells (CCs) support oocyte maturation, and the expansion of CCs contributes to the growth and maturation of the oocyte within the follicle (Turathum et al., 2021). Our study found that FMN effectively promoted the expansion of CCs by increasing the expression of oocyte expansion-related genes, including PTGS2, PTGS1, and CD44. In addition, we also observed an increase in the first polar body extrusion rate (Figure 1). Furthermore, the addition of FMN during the IVC stage significantly enhances the rate of blastocyst formation. Previous studies have indicated that FMN has antioxidant properties, which can effectively eliminate free radicals (Mu et al., 2009). Our study found that during the oocyte period, the expression levels of oocyte redox-related genes, including PGC1α, PRDX2, GPX, and CAT, were significantly higher than those of the control group. Different from the IVC stage, during the embryonic period, the expression levels of SOD1, SOD2, and the oxidative stress-related gene NOX2 were higher. In both periods, FMN significantly reduced ROS and increased GSH levels. Taken together, FMN is capable of exerting antioxidant effects to promote the development of porcine oocytes and embryos *in vitro*.

Mitochondria are the primary site of ROS production in cells and play a crucial role in the oxidative-reductive balance of oocytes and embryos. The physiological processes of oocyte development and early embryo development are notably complex and energy-intensive events (Zhao et al., 2022). Our research showed that, during the oocyte stage, mitochondrial abundance was higher after FMN treatment compared to controls. In the embryonic stage, FMN treatment additionally enhanced mitochondrial membrane potential (MMP) and increased ATP levels (Figure 3). FMN may increase mitochondrial abundance through its antioxidant effect and alleviate the damage of oxidative stress to the mitochondrial membrane, thus stabilizing the membrane potential. Meanwhile, the increase in ATP level directly provides the necessary energy guarantee for the rapid division and differentiation of embryos.

Previous studies have shown that FMN can bind to Keap1 and then change the activity of Nrf2, prompting the dissociation of the Nrf2/Keap1 complex. This enables Nrf2 to translocate to the nucleus, initiate antioxidant components and enhance mitochondrial function (Chen et al., 2023; Yang et al., 2023). In mammals, the Nrf2-Keap1 system serves as an important anti-stress mechanism. Under oxidative stress, Nrf2 is released from Keap1, preventing its ubiquitination and degradation. This leads to the upregulation of antioxidant-related components, enhancement of mitochondrial function, and maintenance of physiological homeostasis (Zhang et al., 2020; Zhang et al., 2013; Fang et al., 2024). Our study found FMN can similarly regulate the Nrf2/Keap1 pathway during the embryonic period. Our research reveals that the expression of Nrf2 is upregulated while that of Keap1 is downregulated under the treatment of FMN. FMN facilitates the nuclear expression of Nrf2, which is confirmed by immunofluorescence and Western blot (Figure 4). Furthermore, at the gene level, FMN also downregulated KEAP1 gene expression and increase NRF2 gene expression. The downstream genes, including NQO-1, SOD1, and SOD2 significant upregulate in response. So we predicted that FMN exerts antioxidant effects to promote embryonic development through the Nrf2/Keap1 pathway.

**FIGURE 7:**
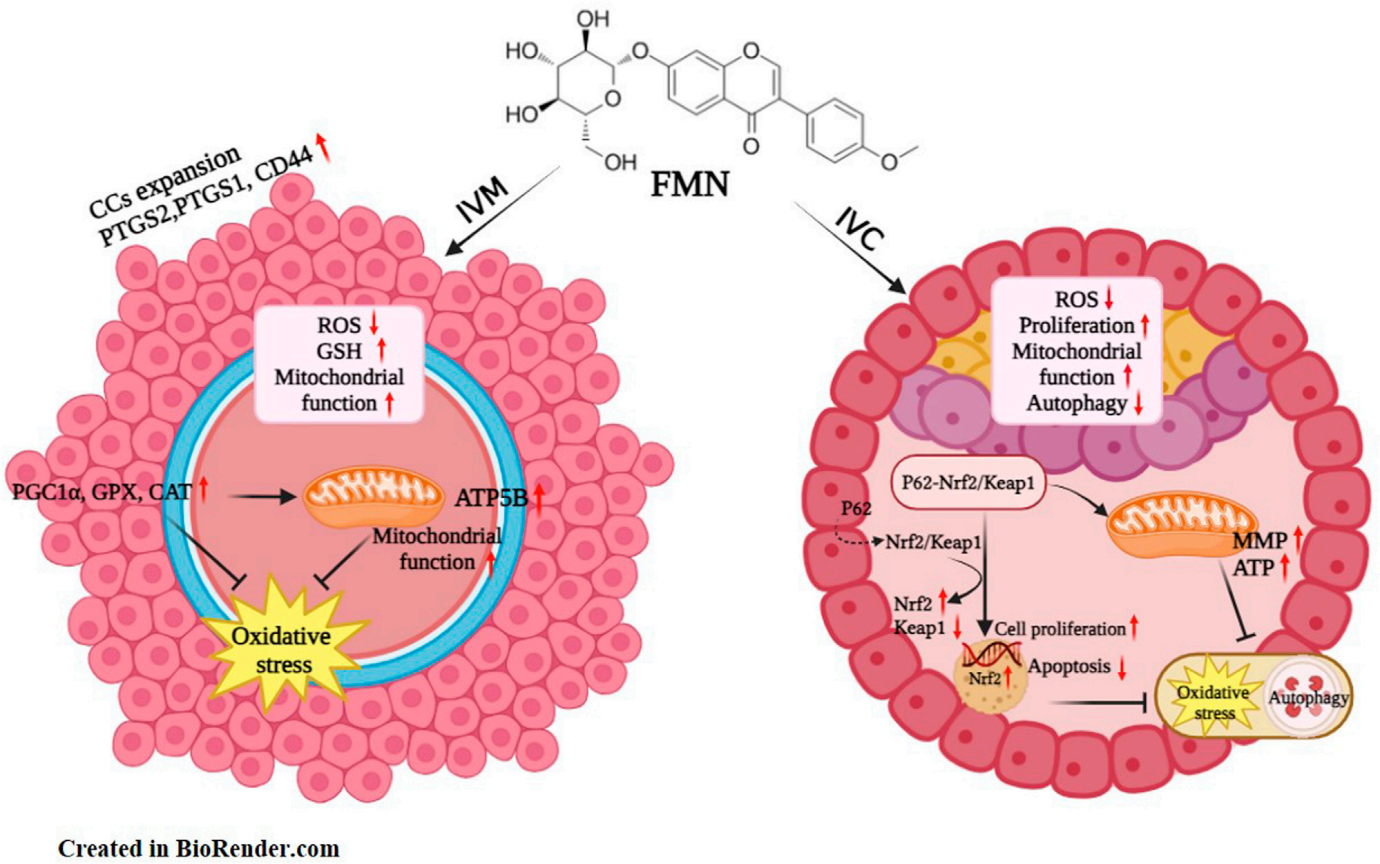
Summary of the effect of Formononetin (FMN) on porcine oocyte *in vitro* maturation and early embryo *in vitro*. This schematic view illustrates that during the IVM stage, FMN enhances oocyte maturation *in vitro* by up-regulating genes related to CCs expansion and antioxidants, reducing oxidative stress, and improving mitochondrial function. During the IVC stage, FMN promotes the nuclear expression of Nrf2 through the P62-Keap1-Nrf2 pathway, which exerts antioxidant function and enhances mitochondrial function. Then, FMN promotes cell proliferation, reduces apoptosis, and lowers autophagy levels.

The activation and modulation of Nrf2 constitute a complex and multifaceted process. Experimental investigations have evinced the crosstalk existing between autophagy and the Nrf2/Keap1 signaling pathway. The adaptor protein P62 can compete with Nrf2 for binding to Keap1, thereby facilitating Nrf2 activation. This activation occurs in response to the reduction of peroxidase activity and the suppression of excessive autophagy as well as ROS generation (Jain et al., 2010; Ichimura et al., 2013; Kong et al., 2021). We examined the impact of FMN on autophagy-related genes in both oocytes and embryos. At the embryonic stage, FMN markedly reduced the protein level of the autophagy marker LC3 (Figure 5). Additionally, it led to a significant decrease in the expression of autophagy-associated genes such as P62, LC3, BECLIN1, and ATG5 (Figure 5). The regulation of oxidative stress levels and autophagy is a dynamically changing process. In this dynamic process, the regulation of the Keap1-Nrf2 pathway by FMN downregulates the levels of oxidative stress and autophagy, thereby promoting the development of oocytes and embryos (Figure 7).

In conclusion, adding the same concentration of FMN to the oocyte and embryo culture media respectively enhanced development and exerted antioxidant effects. Moreover, FMN’s antioxidant mechanism varies between the oocyte and embryonic stages. The potential relationship between such differences in these two phases, like the level of autophagy, requires further exploration.

## Data Availability

The datasets presented in this study can be found in online repositories. The names of the repository/repositories and accession number(s) can be found in the article/Supplementary Material.
