# Supplementary figures and images for: Formononetin promotes porcine oocytes maturation and improves embryonic development by reducing oxidative stress
Source: Front Cell Dev Biol. 2025 Jan 9;12:1520429. doi: 10.3389/fcell.2024.1520429 (PMC11754404; doi:10.3389/fcell.2024.1520429)

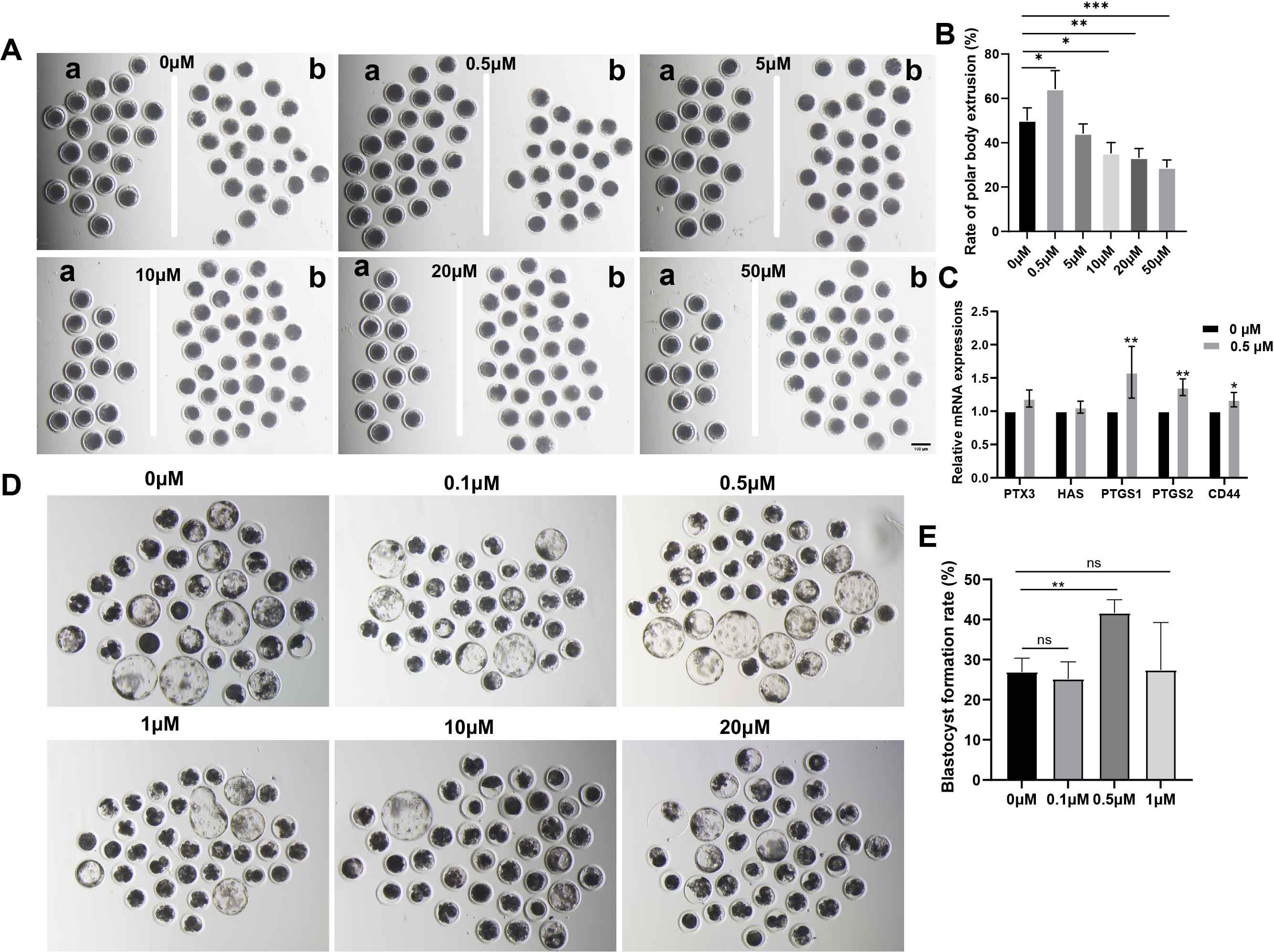

Supplement: Supplementary file 1 [file Image1.JPEG]

## Slide 1
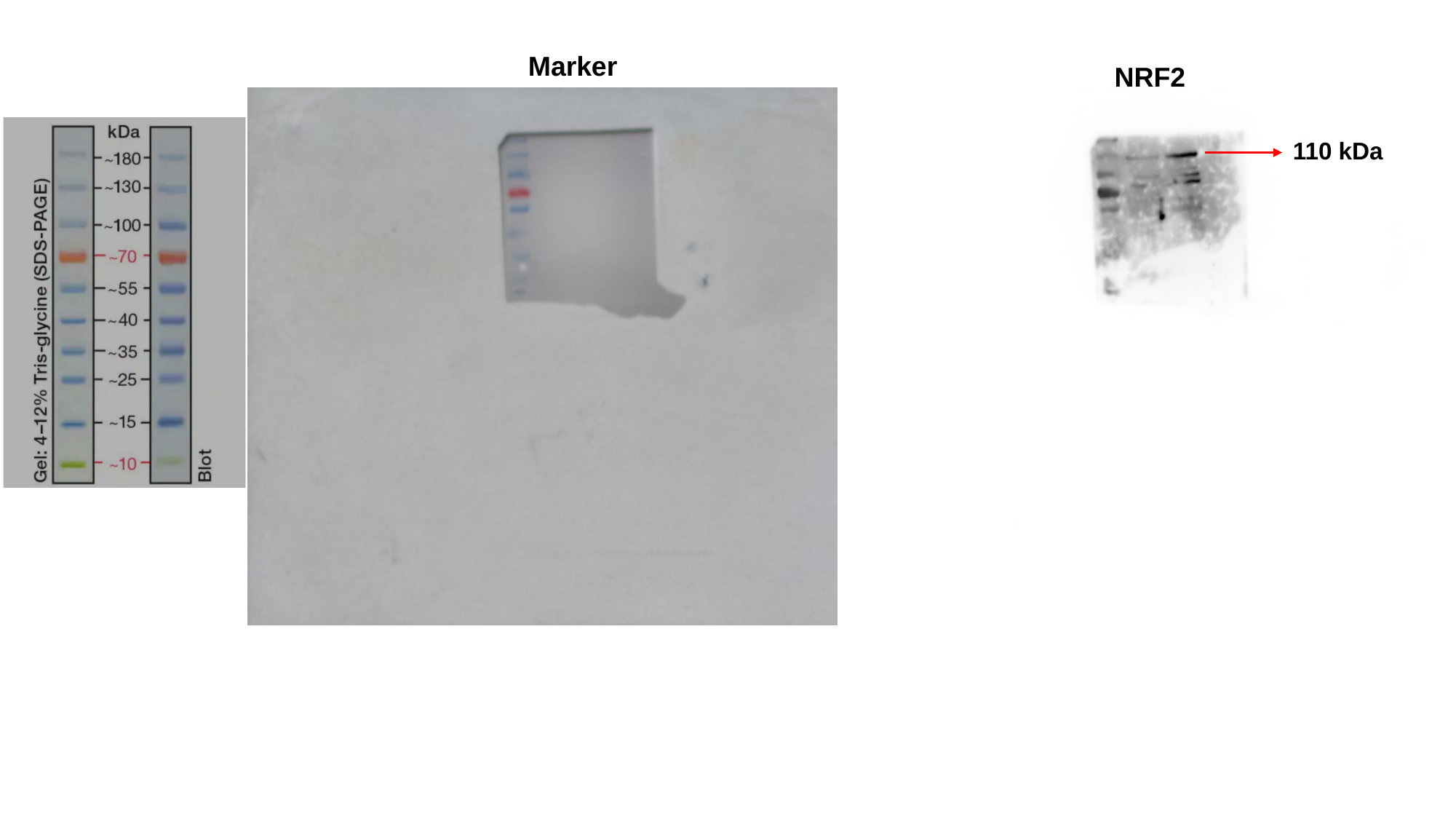

Marker
NRF2
110 kDa

## Slide 2
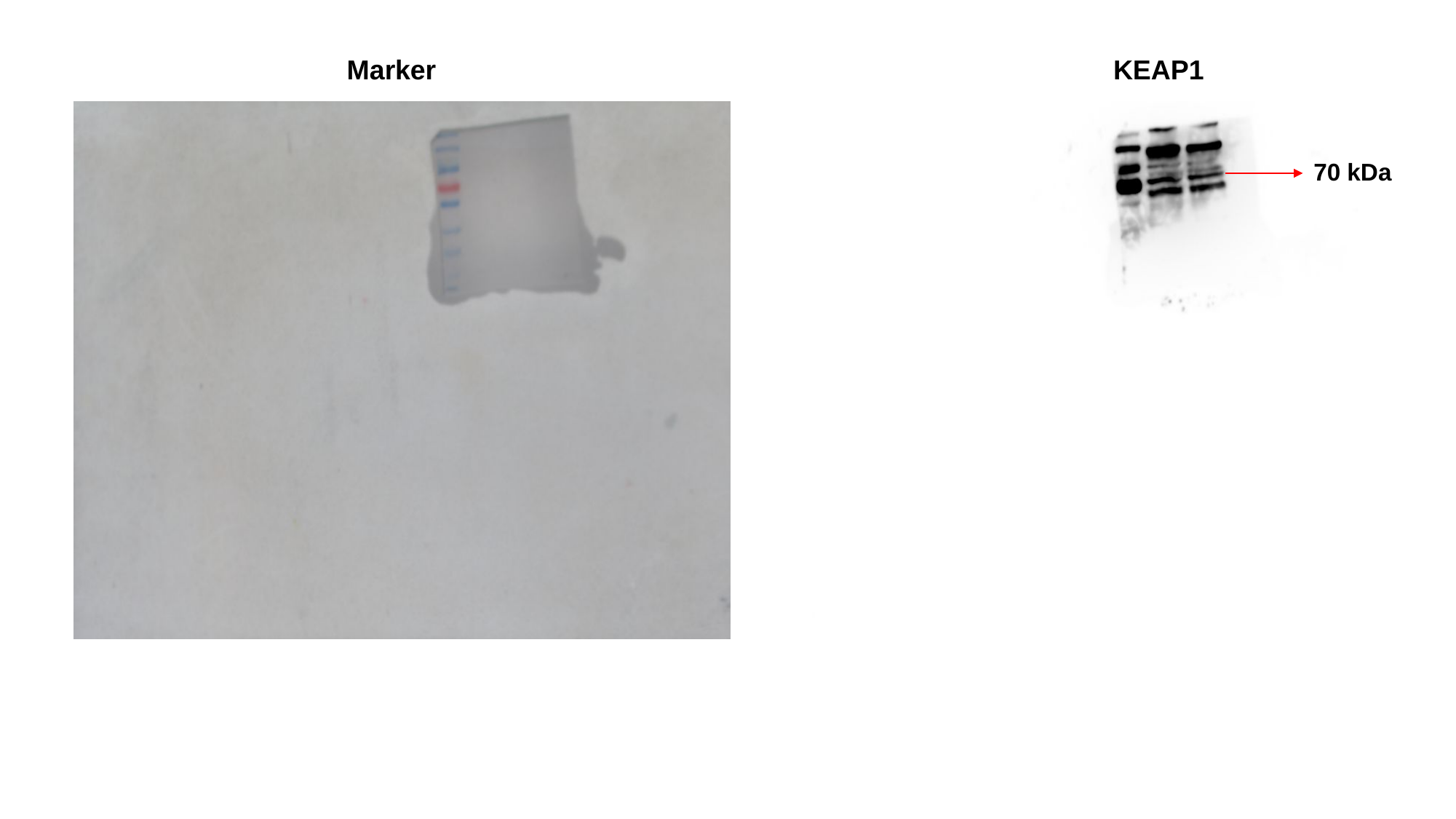

Marker
KEAP1
70 kDa

## Slide 3
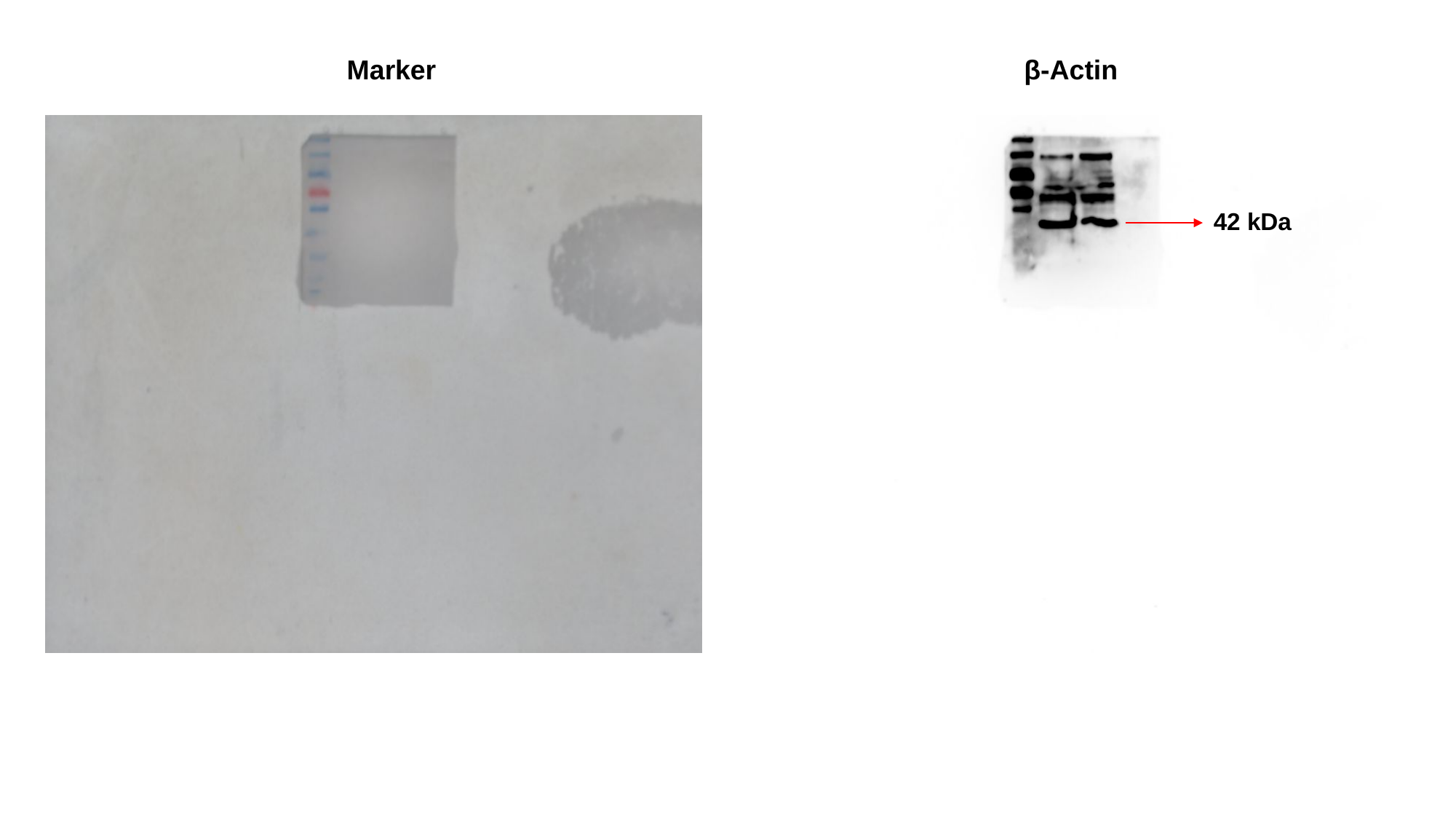

Marker
β-Actin
42 kDa

Supplement: Supplementary file 2 [file DataSheet1.ZIP › full uncropped Gels and Blots images.pptx]
